# Ag Nanorods-Oxide Hybrid Array Substrates: Synthesis, Characterization, and Applications in Surface-Enhanced Raman Scattering

**DOI:** 10.3390/s17081895

**Published:** 2017-08-17

**Authors:** Lingwei Ma, Jianghao Li, Sumeng Zou, Zhengjun Zhang

**Affiliations:** 1 State Key Laboratory of New Ceramics and Fine Processing, School of Materials Science and Engineering, Tsinghua University, Beijing 100084, China; mlw13@mails.tsinghua.edu.cn (L.M.); lijiangh14@mails.tsinghua.edu.cn (J.L.); zousm15@mails.tsinghua.edu.cn (S.Z.); 2 Key Laboratory of Advanced Materials (MOE), School of Materials Science and Engineering, Tsinghua University, Beijing 100084, China

**Keywords:** surface-enhanced Raman scattering (SERS), Ag nanorods-oxide hybrid array substrates, oblique angle vapor deposition (OAD), SERS sensitivity, stability, reusability, qualitative and quantitative analyses, vapor-phase molecule sensing

## Abstract

Over the last few decades, benefitting from the sufficient sensitivity, high specificity, nondestructive, and rapid detection capability of the surface-enhanced Raman scattering (SERS) technique, numerous nanostructures have been elaborately designed and successfully synthesized as high-performance SERS substrates, which have been extensively exploited for the identification of chemical and biological analytes. Among these, Ag nanorods coated with thin metal oxide layers (AgNRs-oxide hybrid array substrates) featuring many outstanding advantages have been proposed as fascinating SERS substrates, and are of particular research interest. The present review provides a systematic overview towards the representative achievements of AgNRs-oxide hybrid array substrates for SERS applications from diverse perspectives, so as to promote the realization of real-world SERS sensors. First, various fabrication approaches of AgNRs-oxide nanostructures are introduced, which are followed by a discussion on the novel merits of AgNRs-oxide arrays, such as superior SERS sensitivity and reproducibility, high thermal stability, long-term activity in air, corrosion resistivity, and intense chemisorption of target molecules. Next, we present recent advances of AgNRs-oxide substrates in terms of practical applications. Intriguingly, the recyclability, qualitative and quantitative analyses, as well as vapor-phase molecule sensing have been achieved on these nanocomposites. We further discuss the major challenges and prospects of AgNRs-oxide substrates for future SERS developments, aiming to expand the versatility of SERS technique.

## 1. Introduction

Since the discovery of surface-enhanced Raman scattering (SERS) in the 1970’s [1], this vibration spectroscopic phenomenon has attracted enormous attention both in experimental study [2,3,4] and theoretical calculation [5,6,7]. Compared with normal Raman signals, the Raman scattering cross-sections of molecules can be enhanced by many orders of magnitude when they are adsorbed on the rough surfaces of noble metal (Au, Ag, and Cu) nanostructures [8,9,10]. This remarkable enhancement is aroused from two types of mechanisms, i.e., electromagnetic (EM) and chemical (CM) enhancements. The EM enhancement originates from the strongly amplified electric field at metal surface that is capable of generating localized surface plasmon resonance (LSPR), which depends significantly on the shape, size, and separation of metallic nanostructures [11,12,13]. The Raman signals of molecules in close proximity to the electromagnetic field are dramatically amplified, with an enhancement factor (EF) of ~10^4^–10^7^ [14,15,16]. While the CM enhancement is caused by the charge transfer between adsorbed molecules and metal surfaces, and always contributes to an EF of 10–10^2^ [17,18,19]. Benefiting from the superior sensitivity and specificity, SERS has been widely implemented in the detection and analysis of molecules at extremely low concentrations, which exhibits tremendous opportunity for biological [20,21], chemical [14,22], clinical [23,24], environmental [25,26], and security sensing applications [27,28].

Recently, nanofabrication approaches based on oblique angle vapor deposition (OAD) [29,30,31] and anodic aluminum oxide (AAO)-templated growth [32,33,34] have been developed to produce uniform and large-area Ag nanorods (AgNRs) arrays as SERS-active substrates. As the combination of OAD and substrate rotation, the glancing angle deposition (GLAD) [35,36] technique is employed to produce well-designed nanostructures such as vertical nanopillars [37], L-shaped AgNRs [38], zigzag columns [39] and spirals [40,41]. Such nanostructures could promote the generation of “hot spots” that are crucial for SERS enhancement. AgNRs substrates with optimal morphology could generate a SERS EF as high as 10^9^ [42], and have been utilized successfully for the determination of chemical molecules [29,33,37], bacteria [43,44], viruses [45,46], amino acids [47], uranyl ion [48], polychlorinated biphenyls [32,34], and so forth. Nevertheless, the development of the SERS technique requires substrates that can not only provide giant enhancement, but also are robust, stable, and easy and relatively inexpensive to fabricate and store. Unfortunately, AgNRs substrates suffer from some intrinsic drawbacks. First, they have a low melting point of ~100 °C [49,50], which causes their thermal instability and as a result, deteriorates their SERS performance at high-temperature conditions. Meanwhile, the highly active surfaces of AgNRs are prone to oxidize/sulfurate in air [51,52,53], and are readily corroded by external etchants [54,55,56], leading to a severe decrease in SERS response. The high costs of SERS detections based on Ag nanostructures also restrict the universality of the SERS technique.

To overcome these inevitable limitations, covering AgNR arrays with thin metal oxide layers has been proposed as a strategy to solve the above problems [49,50,56,57,58,59,60,61]. These oxide materials include Al_2_O_3_, TiO_2_, SiO_2_, HfO_2_, and so on. Taking advantages of the excellent stability and multi-functions of oxide layers, AgNRs-oxide hybrid array substrates provide superior SERS sensitivity and reproducibility, high thermal stability, long-term activity in air, corrosion resistivity, and intense chemisorption of target molecules. These advantages contribute to recyclable and cost-effective SERS substrates for both qualitative and quantitative analyses. This review introduces the synthesis of AgNRs-oxide arrays as versatile SERS substrates, summarizes their structural, physical, and chemical properties, and highlights their practical applications. We further discuss the major challenges and prospects of AgNRs-oxide substrates for future SERS developments.

## 2. Fabrication of AgNRs-Oxide Hybrid Array Substrates

### 2.1. Fabrication of AgNR Arrays

**Figure 1:**
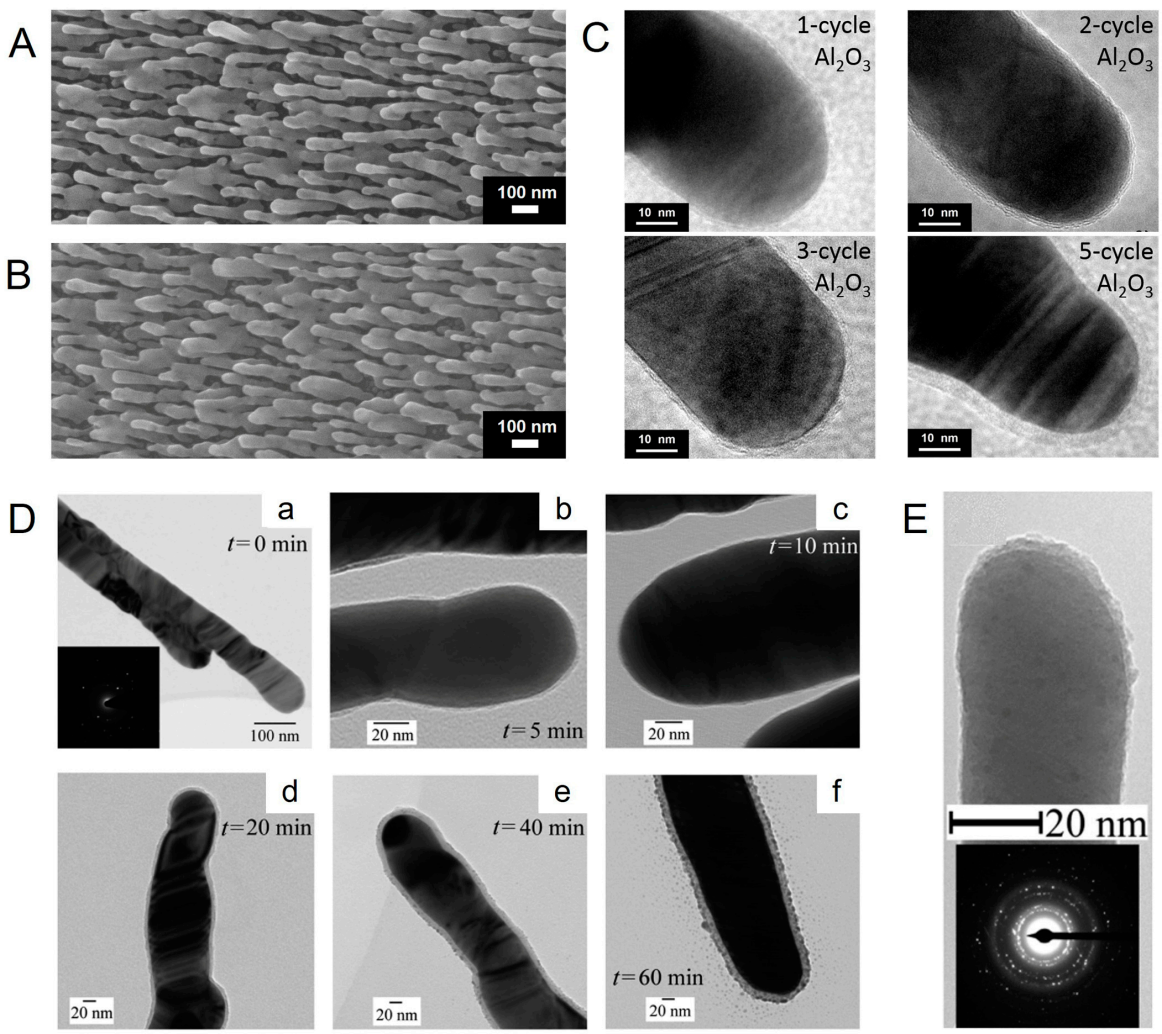
(**A**) Scanning electron microscopy (SEM) image of a pristine AgNRs substrate deposited via the OAD method at the incident angle of 86°, the deposition rate of 0.75 nm/s to a total thickness of 500 nm; (**B**) SEM image of AgNRs coated with an Al_2_O_3_ layer by 5 atomic layer deposition (ALD) cycles; (**C**) Transmission electron microscopy (TEM) images of AgNRs coated with Al_2_O_3_ layers by 1, 2, 3, and 5 ALD cycles, respectively (Reprinted with permission from [49]); (**D**) TEM images of AgNRs before and after coating with SiO_2_ layers for different reaction times (Reprinted with permission from [58]. Copyright (2011) American Chemical Society); (**E**) TEM image of a single AgNR with TiO_2_ capping primarily at the nanorod tip (top), and electron diffraction pattern of multiple AgNRs (bottom) (Reprinted with permission from [50]. Copyright (2014) AIP Publishing LLC).

AgNR arrays are synthesized based on OAD technique. OAD is a physical vapor deposition technique in which the vapor atoms are deposited at a large incident angle *θ* (>70°) with respect to the substrate normal [31,37]. The growth of AgNRs is controlled by shadowing effect and surface diffusion, and their morphology can be readily tailored by tuning the deposition conditions such as incident angle, growth time, growth rate, and substrate temperature. Typically, AgNRs were prepared on Si wafers in an electron-beam system at high vacuum level. During deposition, the incident angle of vapor flux was set at ~86° off the substrate normal. The deposition rate was fixed at 0.75 nm/s to a desirable thickness read by a quartz crystal microbalance (QCM) [49,57]. As shown in Figure 1A, the resulted AgNRs are of cylindrical shape, uniformly distributed and well-separated. The detailed deposition procedure can be found in previous publications [30,31,37,58].

### 2.2. Fabrication of AgNRs-Oxide Hybrid Array Substrates with Different Oxide Layers

Several approaches have been exploited to produce oxide layers over AgNR arrays. Because SERS is a highly localized effect that depends significantly on the distance between metal surfaces and target molecules [62,63,64], the oxide shells should be thin enough so as not to eliminate the SERS enhancement. In this regard, atomic layer deposition (ALD) holds great potential in the oxide formation of AgNRs-oxide hybrid substrates. ALD is a unique thin film growth technique by means of sequential self-limiting surface reactions of gaseous precursors [65,66,67]. It is capable of preparing high-quality films with precise thickness control at the atomic scale, excellent conformality independent of the substrate geometry, low defect density, and large-scale uniformity. As an example, Ma et al. have deposited Al_2_O_3_ over AgNRs using trimethylaluminum (TMA) and water as ALD precursors [49]. High purity N_2_ was adopted as the carrier and purge gas. Typically, one complete reaction cycle consisted of four steps: (1) TMA reactant exposure; (2) N_2_ gas purging; (3) water vapor exposure; and (4) N_2_ gas purging. This reaction cycle was repeated for different times so as to control the Al_2_O_3_ thickness. TiO_2_ and HfO_2_ have also been successfully deposited over AgNRs by ALD [57,60], with similar reaction cycles but different precursors and reaction time. It is worth noticing that a low-temperature ALD process at 50 to 80 °C is required to avoid the coarsening and fusion of the underneath AgNR arrays during oxide growth. Besides, based on the hydrolysis reaction of tetraethyl orthosilicate (TEOS), uniform and conformal SiO_2_ layers have been coated onto AgNRs to form AgNRs-SiO_2_ core-shell nanostructures [58]. The shell growth made porous SiO_2_ more compact, and its thickness was tailored by varying the coating time. Moreover, Huang et al. proposed to cover AgNRs with a thin TiO_2_ layer directly through OAD fabrication [50]. To be specific, after AgNRs deposition, they changed the evaporation material from Ag to TiO_2_, and did the deposition again at the same substrate orientation. Because this coating approach only requires the OAD system instead of many complicated apparatuses, it is very efficient and cost-effective and thus promising for real-world fabrication.

## 3. Characterization of AgNRs-Oxide Hybrid Array Substrates

### 3.1. Morphology of AgNRs-Oxide Hybrid Array Substrates

Figure 1B shows the scanning electron microscope (SEM) image of the AgNRs-oxide substrate. Due to the ultrathin feature of oxide layer, AgNRs-oxide arrays reveal no visible morphology variation compared with the pristine AgNRs. To have a better observation of the oxide shells, high-resolution transmission electron microscope (HRTEM) analysis is employed to provide a visual evidence, which is also applied to investigate oxide thickness growth. As illustrated in Figure 1C,D, the oxide layers fabricated by ALD and hydrolysis reaction are amorphous in structure and of different thickness, uniformly and conformally wrapping AgNRs. When the reaction time is very short, the as-prepared oxide layers possess a few pinholes; as the reaction continues, the shells become thicker and more compact. HRTEM results also demonstrate that the growth thickness increases linearly with the ALD cycle/reaction time [49,58], which is beneficial for us to precisely control the layer thickness to sub-nanometer scale. The thickness of Al_2_O_3_, TiO_2_, and HfO_2_ shells fabricated by ALD is about 0.6–0.8 nm per ALD cycle [49,57,60], and that of SiO_2_ grown by hydrolysis reaction is approximately 0.27 nm/min [58]. For TiO_2_ capping prepared by the OAD method in Figure 1E, it is about 5 nm thick and mainly located on the top surfaces of AgNRs, and has both amorphous and crystalline regions [50].

### 3.2. SERS Sensitivity and Reproducibility of AgNRs-Oxide Hybrid Array Substrates

**Figure 2:**
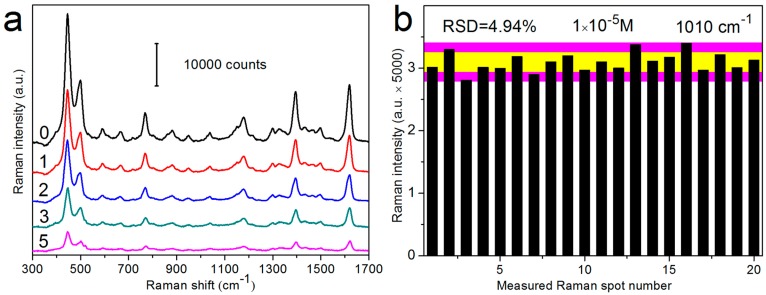
(**a**) Raman spectra of 5 × 10^−6^ M methylene blue (MB) molecules adsorbed on the uncoated AgNRs substrate (0 cycle) and on AgNR arrays coated with Al_2_O_3_ layers by 1, 2, 3, and 5 ALD cycles (Reprinted with permission from [49]); (**b**) SERS intensity distribution of 1 × 10^−5^ M dipicolinic acid (DPA) at 1010 cm^−1^ band from 20 randomly selected spots over the AgNRs substrate coated with an Al_2_O_3_ layer by 1 ALD cycle (Reprinted with permission from [56]. Copyright (2015) American Chemical Society).

It has been long recognized that the SERS enhancement of metal nanostructures depends strongly on the distance between metal surfaces and adsorbed molecules. We therefore investigate the coating effect on the sensitivity of SERS substrates. The results in Figure 2a clearly present that the SERS efficiency of AgNR arrays decreases to ~65% and ~50% after ~0.7 nm (1 ALD cycle) and ~1.5 nm (2 ALD cycles) oxide coating, and declines monotonously with further increasing the oxide thickness [49]. We should note that, due to the ultrathin feature of oxide layers, the strong SERS enhancement of AgNRs is well maintained. All hybrid substrates exhibit satisfactory SERS EFs on the order of 10^7^ [49,57,60], confirming the remarkable sensitivity of AgNRs-oxide nanocomposites. A low relative standard deviation (RSD) value with Raman signals of ~5% [56] (see Figure 2b) indicates that the AgNRs-oxide substrates are uniform in structure and of good reproducibility for SERS measurements, which is also a prerequisite for quantitative analysis.

### 3.3. Thermal Stability of AgNRs-Oxide Hybrid Array Substrates

**Figure 3:**
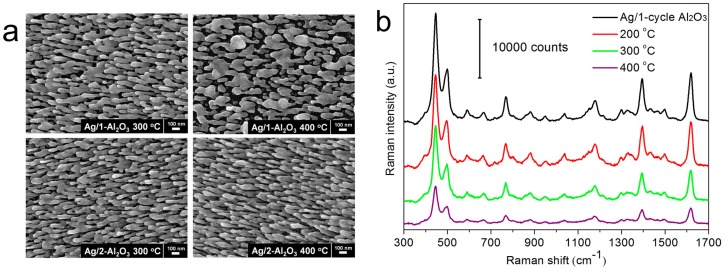

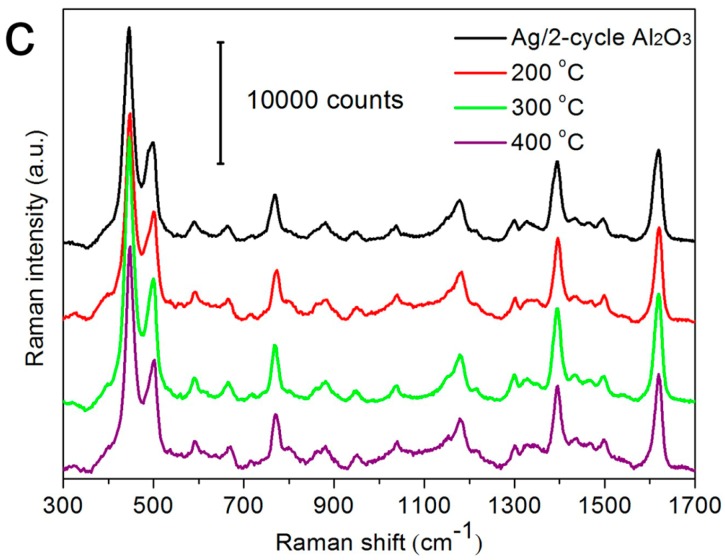
(**a**) SEM images of AgNR arrays coated with Al_2_O_3_ layers by 1 and 2 ALD cycles after annealing at 300 and 400 °C; Raman spectra of 5 × 10^−6^ M MB adsorbed on AgNRs substrates coated with Al_2_O_3_ layers by (**b**) 1 ALD cycle and (**c**) 2 ALD cycles, before/after annealing at 200, 300, and 400 °C, respectively (Reprinted with permission from [49]).

High-temperature SERS detection is a vital part for routine applications, which can be utilized for monitoring many in situ reactions, such as thermal crystallization [68], structural variations [69,70], and chemical reactions [52,71] at elevated temperatures. For pristine AgNR arrays, their structure begins to change at a very low temperature of 50 °C, and collapses completely at 100 °C [49,50]. It is thereby highly demanded to improve the thermal stability of AgNRs-based sensors both in morphology robustness and SERS sensitivity. Since the melting point of oxides (1700–2700 °C) is much higher than that of silver (960 °C), covering AgNR arrays with oxide layers might be effective to address this issue. It is illustrated in Figure 3a that the AgNRs substrate coated by 1-cycle Al_2_O_3_ (~0.7 nm) are robust in morphology at 200 °C, but melt partly at 300 and 400 °C. For AgNRs coated by 2-cycle Al_2_O_3_ of ~1.5 nm thick, no obvious structural change is observed after being heated at 300 and 400 °C [49]. The morphology robustness also leads to the stabilized SERS performance at elevated temperatures, demonstrated in Figure 3b,c. As such, AgNR arrays coated with ~1.6 nm TiO_2_ or HfO_2_ shell also sustain their morphology and SERS efficiency at 300–400 °C [57,60]. Moreover, by capping the top surfaces of AgNRs with high melting-temperature TiO_2_ of ~5 nm, the Ag mass transport from tips to sides is slowed down. They preserve their shapes well at 100 °C, while they coarsen to some extent at 200 °C [50]. These results suggest that the oxide coating/capping functions as a barrier to protect AgNRs both in morphology stiffness and SERS sensitivity against high temperatures, and covering the entire surfaces of AgNRs is especially useful to achieve these goals.

### 3.4. Temporal Stability of AgNRs-Oxide Hybrid Array Substrates

**Figure 4:**
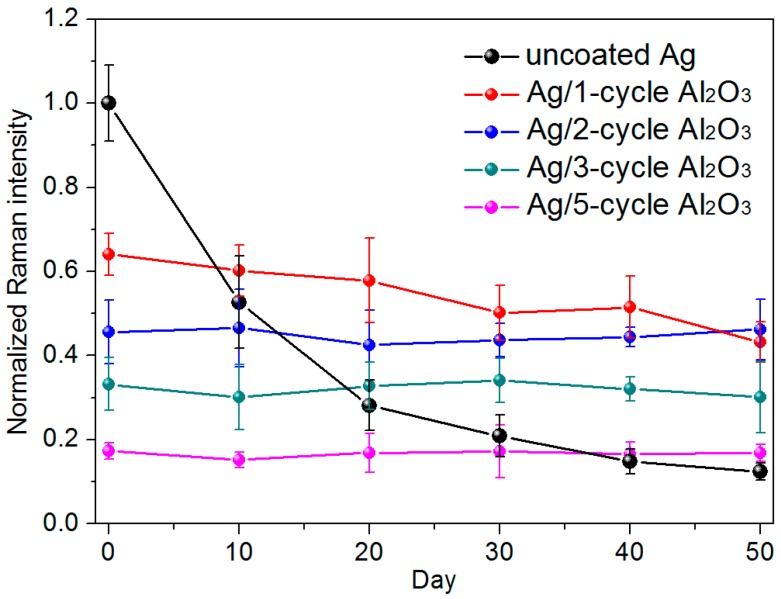
The normalized Raman intensities of 5 × 10^−6^ M MB Raman peak at 1622 cm^−1^ on the uncoated AgNRs substrate and on AgNR arrays coated with Al_2_O_3_ layers by 1, 2, 3, and 5 ALD cycles, as a function of the measurement time (Reprinted with permission from [49]).

The effect of oxide coating on the temporal stability of AgNRs in air is investigated as a function of shelf time, see details in Figure 4 [49]. For pristine AgNR arrays, due to the highly active surfaces, the SERS activity drops drastically in air, which declines by half after 10-day storage time and is about one order smaller after 50 days. On the other hand, when AgNRs are uniformly wrapped with oxides, the protective shells could suppress their surface reactions with air, and accordingly the shelf life is dramatically increased. Specifically, the AgNRs substrate coated with ~0.7 nm Al_2_O_3_ presents a slight signal decrease after 50 days, which can be explained by the pinhole-containing feature of the ultrathin oxide shell. While the substrates coated by ~1.5 nm or thicker Al_2_O_3_ remain almost constant in SERS response during the whole test period. As a result, a thin but compact coating layer could sufficiently passivate the internal Ag NRs so as to stabilize their SERS activity under atmospheric conditions for a long period. Similar conclusion has been verified on AgNRs-TiO_2_ substrates [57].

### 3.5. Chemical Stability of AgNRs-Oxide Hybrid Array Substrates

**Figure 5:**
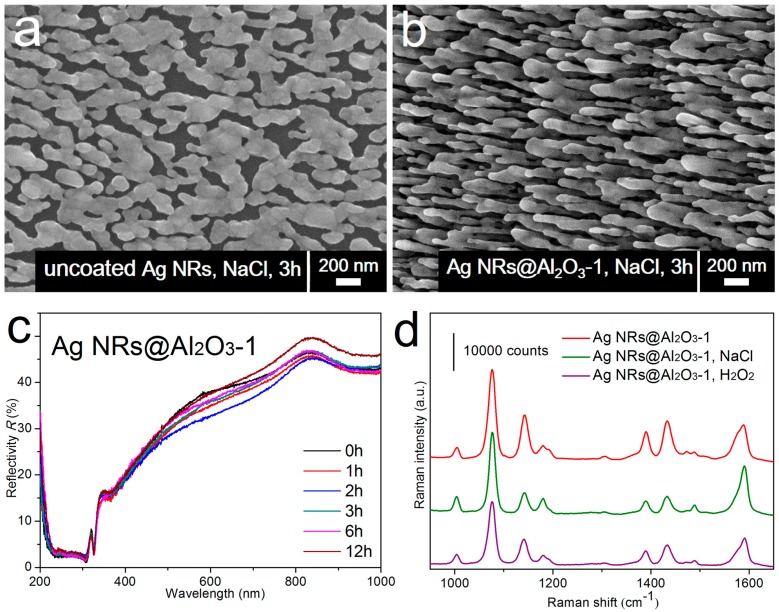
SEM images of (**a**) pristine AgNRs and (**b**) the AgNRs substrate coated with 1-cycle Al_2_O_3_ after being merged in a 30 mM NaCl solution for 3 h; (**c**) reflectance spectra variations of the AgNRs substrate coated with 1-cycle Al_2_O_3_ within a 12 h NaCl erosion time; (**d**) SERS performance of AgNRs coated with 1-cycle Al_2_O_3_ before/after NaCl (30 mM, 3 h) and H_2_O_2_ (2.2%, 0.5 h) immersion, using 1 × 10^−6^ M 4-aminothiophenol (4-ATP) as probing molecules (Reprinted with permission from [56]. Copyright (2015) American Chemical Society).

To function as reliable SERS sensors, the chemical stability is of essential importance especially at erosive environments. Because chloride ions [54,72,73], strong oxidants [54,56], and acidic solutions [61,74] can severely etch silver atoms and cause the shape transformation of Ag nanostructures (see Figure 5a as an example), it is crucial to improve the chemical stability of AgNRs against corrosion. We find that after Al_2_O_3_ deposition, the chemical stability of coated substrates is substantially improved, even if the oxide shell is sub-nanometer and has a few pinholes [56]. As shown in Figure 5b–d, the chemically inert Al_2_O_3_ shell prevents the internal AgNRs from direct contact with external etchants such as NaCl and H_2_O_2_, retaining sufficiently their morphology and SERS efficiency. Additionally, for strong acidic media where Al_2_O_3_ will dissolve quickly, ultrathin HfO_2_ film has been implemented to protect AgNRs from failure [61]. This kind of substrate possesses the acid-resistant property, making it applicable in acid solutions of practical environments.

## 4. Applications of AgNRs-Oxide Hybrid Array Substrates

### 4.1. Reusable SERS Substrates

**Figure 6:**
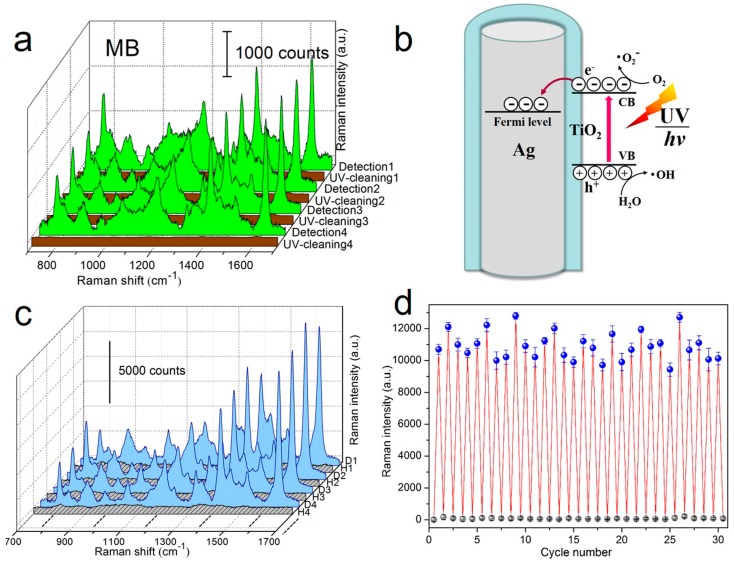
(**a**) Raman spectra of 5 × 10^−6^ M MB molecules adsorbed on the AgNRs substrate coated with ~2 nm TiO_2_ in four “detection-UV cleaning” cycles; (**b**) the schematic for the photocatalytic mechanism of AgNRs-TiO_2_ hybrids (Reprinted with permission from [57]); (**c**) Raman spectra of 1 × 10^−6^ M MB on the AgNRs substrate coated with ~1.6 nm HfO_2_ measured in multiple “detection-heating” cycles and (**d**) the 1623 cm^−1^ peak intensity variations in 30 cycles (Reprinted with permission from [60]. Copyright (2016) American Chemical Society).

Given that SERS substrates are generally made of noble metals and are not readily reused, the costly preparation and disposable property seriously hinder the universality of SERS technique. For this reason, it is significant to develop recyclable SERS substrates. One feasible way is the direct degradation of adsorbed molecules from substrate surfaces after SERS identification. For AgNR arrays coated with photocatalytic material TiO_2_ [75,76,77], the self-cleaning ability is realized through ultraviolet (UV) light-induced decomposition of organic molecules adsorbed on the substrate, i.e., subsequent to SERS measurements, the substrate can be purified by UV illumination and be reused for further SERS analysis [57]. As revealed in Figure 6a, four “detection-UV cleaning” circulations are carried out on the AgNRs substrate coated with ~2 nm TiO_2_. Strong target signals are observed during SERS sensing, and they almost vanish after UV irradiation. More importantly, the results from the subsequent three circulations show that the Raman intensities are fully recovered at each detection step, which suggests that the AgNRs-TiO_2_ structure is capable of enduring multiple UV irradiations with considerable robustness. Another point to note is that the molecule degradation capability not only comes from the photocatalysis of TiO_2_, it also benefits from the intensive interaction between Ag and TiO_2_ that optimizes the separation of photo-excited charge carriers and as a result facilitates the degradation efficiency (see Figure 6b) [78,79,80].

Thermal annealing is another way to detach molecules from adsorbed surfaces, and accordingly it might be helpful to clean and regenerate SERS substrates. By virtue of the high melting-temperature HfO_2_ [81], the AgNRs substrate coated by ~1.6 nm HfO_2_ represent good thermal stability and morphological robustness at temperatures up to 400 °C [60]. After SERS detection, the regeneration of AgNRs-HfO_2_ can be achieved by heating the substrate on a hot plate within several seconds. This process leads to the thermal release of adsorbed molecules and refreshes the substrate for subsequent measurements. From Figure 6c,d, one sees that the hybrid substrate maintains its SERS efficiency well during 30 “detection-heating” cycles, demonstrating the remarkable stability and recyclability of AgNRs-HfO_2_ substrate. As a consequence, reusability could eliminate the single-use shortcoming of conventional SERS substrates, in which way the high costs for SERS measurements are substantially reduced and the practicability of the SERS technique is extended.

### 4.2. Qualitative and Quantitative SERS Analyses

**Figure 7:**
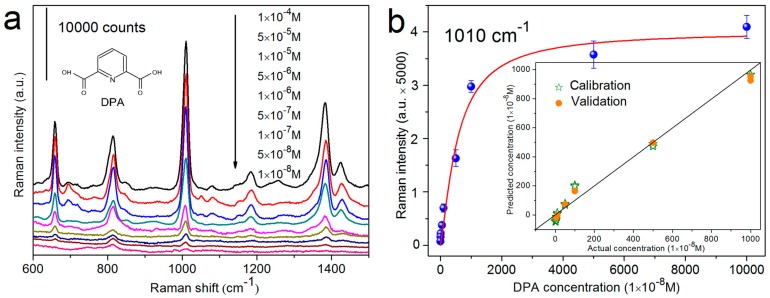
(**a**) SERS spectra of dipicolinic acid (DPA) on the AgNRs substrate coated with an Al_2_O_3_ layer by 1 ALD cycle, with concentrations from 1 × 10^−4^ to 1 × 10^−8^ M; (**b**) The concentration dependence of DPA peak intensity at 1010 cm^−1^ as a function of DPA concentrations ranging from 1 × 10^−4^ to 1 × 10^−8^ M. The inset illustrates the actual DPA concentrations versus their predicted values between 1 × 10^−5^ to 1 × 10^−8^ M with the partial least squares regression (PLSR) model (Reprinted with permission from [56]. Copyright (2015) American Chemical Society).

To make SERS technique a practical and reliable analysis tool, both qualitative and quantitative abilities are required for desirable SERS substrates. It has been reported that Al_2_O_3_ has high affinity to carboxyl (–COOH) functional groups, ascribed to the strong polar interaction [82,83]. Therefore, we adopt AgNRs-Al_2_O_3_ substrates to enhance the absorbability and correspondingly SERS detection efficiency of carboxylic acids. Dipicolinic acid (DPA) is a commonly used biomarker for the recognition of bacterial spores [83,84], so its sensitive and quantitative probing is particularly important. Figure 7a shows the Raman spectra of DPA by employing AgNRs coated with 1-cycle Al_2_O_3_ as the SERS platform, with the limit of detection (LOD) down to 10^−8^ M. Meanwhile, the partial least squares regression (PLSR) [85,86] model in Figure 7b exhibits good predictability within the concentration ranging from 1 × 10^−8^ to 1 × 10^−5^ M, which provides a calibration for the quantification of trace DPA. In addition to analytes that can directly react with Al_2_O_3_ surfaces, for those who have no or weak interaction with oxides (such as pyridine, acridine, and cyanide [87,88,89]), the pinhole-containing AgNRs-Al_2_O_3_ arrays provide a channel to anchor them directly onto Ag surfaces through Al_2_O_3_ pinholes. For instance, the LOD of NaCN on the pinhole-containing AgNRs-Al_2_O_3_ substrate is as low as 1 ppb, and quantitative analysis also reveals a satisfactory PLSR predictability [56]. These results suggest that AgNRs-oxide substrates are appropriate to detect a variety of molecules in both qualitative and quantitative manners.

**Figure 8:**
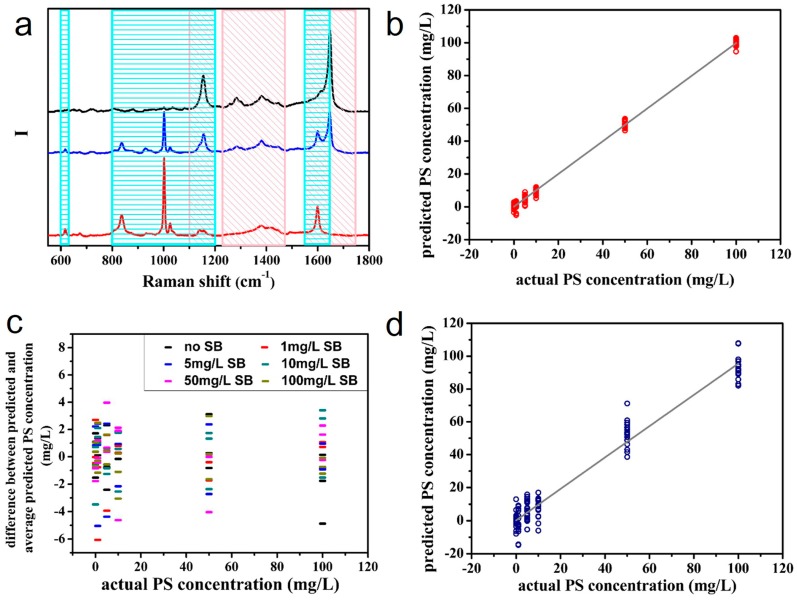
(**a**) The black, blue, and red lines represent the Raman spectra of PS solution, mixture solution containing PS and SB molecules, and SB solution measured with SERS substrates, respectively; the pink and blue rectangles mark the characteristic peaks of PS and SB that are employed to calibrate PLSR model; (**b**) PS concentration predicted by the PLSR model established with Raman spectra corresponding to PS characteristic peaks; (**c**) difference between predicted PS concentration of each mixture and average predicted PS concentration of solution with the same PS concentration; (**d**) PS concentration in the test solution with different compositions predicted by the PLSR model of PS (Reprinted with permission from [61]).

From a more realistic perspective, AgNRs-oxide substrates have been adopted to sense food antiseptics [61]. It is known that food antiseptics with appropriate amount could inhibit bacteria and extend food’s shelf life; whereas excessive addition might be harmful for human health [90,91]. Hence, quantitative analysis of antiseptics based on SERS is of great value. Potassium sorbate (PS) and sodium benzoate (SB) are common food antiseptics. Because they only work in acidic media [92,93], the AgNRs-HfO_2_ substrate with acid resistance is applicable for their identification [61]. The LODs of these two antiseptics are both 300 μg/L, which are much lower than their dosage standard in food. Therefore, the SERS substrate meets the demand of identifying PS and SB in practice. Moreover, the PLSR relationship between the concentrations and SERS spectra of a series of PS solutions performs quite well. As for the mixture of PS and SB, even if some of their characteristic peaks overlap, the two respective PLSR models of them are both accurate and reliable (see Figure 8). That is to say, the Raman peaks corresponding to SB in spectra do not interfere the quantification of PS molecules, and vice versa.

### 4.3. Vapor-Phase Molecule Sensing

**Figure 9:**
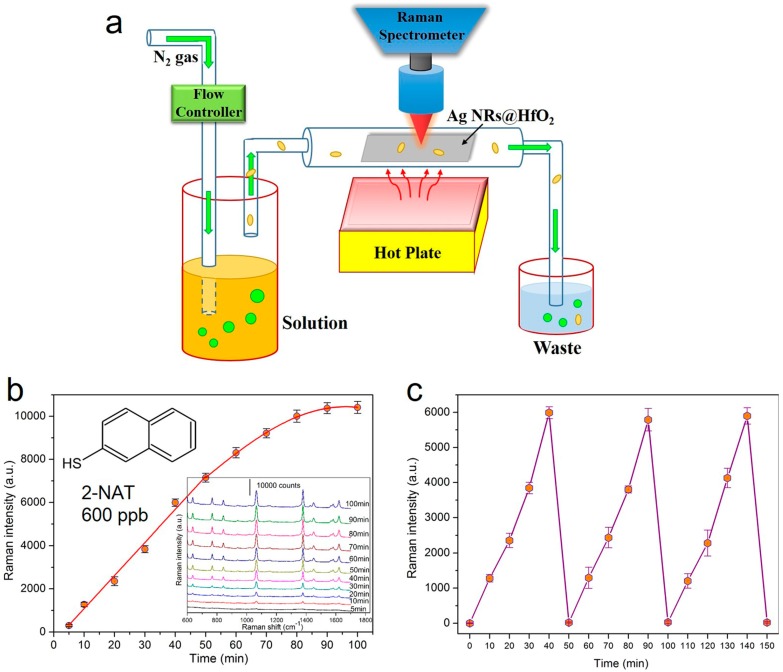
(**a**) Schematic of the gas sensing device; (**b**) SERS spectra and the 1379 cm^−1^ peak intensity of 600 ppb 2-NAT on the AgNRs-HfO_2_ substrate as a function of gas flow time. (**c**) 1379 cm^−1^ peak intensity variations of 600 ppb 2-NAT during the repetition of “vapor exposure-thermal cleaning” cycles on the substrate (Reprinted with permission from [60]. Copyright (2016) American Chemical Society).

Aside from revealing the feasibility of SERS determination in liquids, AgNRs-oxide arrays also provide real-time monitoring of vapor-phase molecules at ultralow concentrations [60]. To capture and sense target gases, the AgNRs-HfO_2_ substrate is placed in a homemade gas detection system presented in Figure 9a. 2-naphthalenethiol (2-NAT) is selected as the model gas, and high purity N_2_ is utilized as the carrier gas. During gas detection, N_2_ is injected into the analyte solution, and target molecules in the vapor phase are carried out together with the N_2_ flow and are captured by the AgNRs-HfO_2_ platform. SERS spectra are recorded simultaneously during gas passing. It is shown in Figure 9b that the 2-NAT signals ascend continuously with the gas flowing until the saturation of the substrate surface. The LOD of 2-NAT is down to 20 ppb, verifying the effectiveness of AgNRs-HfO_2_ substrate for gas detection. Furthermore, we explore the renewability of AgNRs-HfO_2_ arrays during gas recognition, i.e., after SERS measurement, the substrate is heated on a hot plate at 250 °C for 30 s to desorb molecules. The results in Figure 9c reveal that the Raman intensities of 600 ppb 2-NAT at 1379 cm^−1^ peak escalate with vapor passing and after each annealing treatment, the substrate is free of 2-NAT while it is totally recovered in the following “vapor exposure-thermal cleaning” cycles. We believe this highly robust and versatile SERS platform could act as a recyclable sensor for in situ monitoring of complex gases from realistic environments, such as air pollutants [94,95], explosives [96,97], volatile organic compounds [98,99], and chemical warfare agents [100,101].

## 5. Conclusions

This review provides insights for the synthesis, characterization, and applications of AgNRs-oxide hybrid array substrates as SERS sensing platforms, all in order to emphasize our understanding and utilization of the SERS technique. AgNR arrays are prepared based on the OAD method, and oxide shells are readily coated onto AgNRs by diverse approaches with controllable thickness and excellent uniformity. The characterization of these nanocomposites is detailed in aspects of shell growth, SERS sensitivity, and reproducibility, as well as thermal, temporal, and chemical stability. By virtue of the ultrathin thickness, uniformity, and stability of oxide layers, AgNRs-oxide substrates possess large SERS EFs, outstanding reproducibility, and excellent stability. In addition, the reusability, quantification, and gas sensing have been achieved on the substrates, which hold great potential for the identification of trace analytes in real systems. 

Although AgNRs-oxide array substrates have proved their extraordinary advantages, they are still in their infancy for practical SERS applications, and several challenges are yet to be addressed. First, AgNRs-oxide hybrid arrays possess satisfactory reproducibility and reliability exceeding the performance of commonly used nanoparticles, which are crucial for biosensing. The qualitative and quantitative detection of DPA, an anthrax biomarker, has been achieved on the AgNRs-Al_2_O_3_ substrate. As for the SERS-based immunoassay and nucleic acid detection, AgNRs-oxide arrays offer potent capture substrates to bind SERS nanotags. The close proximity of the substrate–nanotag interface provides additional plasmonic coupling that further increases the SERS enhancement. It is thus urgent to demonstrate the SERS determination of bacteria, viruses, nucleic acids, and proteins on AgNRs-oxide arrays before the substrates can be routinely applied. Second, surface functionalization [102,103,104] should be realized on AgNRs-oxide substrates to further boost their widespread employments. Third, because different oxide materials possess various unique features according to their chemical or physical properties, further exploration of the diverse functions of AgNRs-oxide nanocomposites is still in great demand, which is essential for developing multifunctional SERS sensors in certain conditions. Moreover, aside from oxides, combining AgNR arrays with other attractive materials, e.g., graphene [105,106,107] and MoS_2_ [108,109], is another advisable method for future SERS developments. Overall, along with the growing study, deeper understanding, and better optimization of AgNRs-oxide nanostructures, these substrates hold great promise as affordable and portable SERS sensors, and would open up a new era for practical SERS applications.

## References

[1] Fleischmann M., Hendra P.J., McQuillan A.J. (1974). Raman spectra of pyridine adsorbed at a silver electrode. Chem. Phys. Lett..

[2] Xu L., Yan W., Ma W., Kuang H., Wu X., Liu L., Zhao Y., Wang L., Xu C. (2015). SERS encoded silver pyramids for attomolar detection of multiplexed disease biomarkers. Adv. Mater..

[3] Li J., Liu J., Yang Y., Qin D. (2015). Bifunctional Ag@Pd-Ag nanocubes for highly sensitive monitoring of catalytic reactions by surface-enhanced Raman spectroscopy. J. Am. Chem. Soc..

[4] Guo P., Sikdar D., Huang X., Si K.J., Xiong W., Gong S., Yap L.W., Premaratne M., Cheng W. (2015). Plasmonic core-shell nanoparticles for SERS detection of the pesticide thiram: Size-and shape-dependent Raman enhancement. Nanoscale.

[5] Wang X., Li M., Meng L., Lin K., Feng J., Huang T., Yang Z., Ren B. (2013). Probing the location of hot spots by surface-enhanced Raman spectroscopy: Toward uniform substrates. ACS Nano.

[6] Wang T., Zhang Z., Liao F., Cai Q., Li Y., Lee S., Shao M. (2014). The effect of dielectric constants on noble metal/semiconductor SERS enhancement: FDTD simulation and experiment validation of Ag/Ge and Ag/Si substrates. Sci. Rep..

[7] Huang Y., Ma L., Hou M., Xie Z., Zhang Z. (2016). Gradual plasmon evolution and huge infrared near-field enhancement of metallic bridged nanoparticle dimers. Phys. Chem. Chem. Phys..

[8] Fu Q., Zhan Z., Dou J., Zheng X., Xu R., Wu M., Lei Y. (2015). Highly reproducible and sensitive SERS substrates with Ag inter-nanoparticle gaps of 5 nm fabricated by ultrathin aluminum mask technique. ACS Appl. Mater. Interfaces.

[9] Lu R., Konzelmann A., Xu F., Gong Y., Liu J., Liu Q., Xin M., Hui R., Wu J.Z. (2015). High sensitivity surface enhanced Raman spectroscopy of R6G on *in situ* fabricated Au nanoparticle/graphene plasmonic substrates. Carbon.

[10] Li D., Liu J., Wang H., Barrow C.J., Yang W. (2016). Electrochemical synthesis of fractal bimetallic Cu/Ag nanodendrites for efficient surface enhanced Raman spectroscopy. Chem. Commun..

[11] Nie S., Emory S.R. (1997). Probing single molecules and single nanoparticles by surface-enhanced Raman scattering. Science.

[12] Xu H., Aizpurua J., Käll M., Apell P. (2000). Electromagnetic contributions to single-molecule sensitivity in surface-enhanced Raman scattering. Phys. Rev. E.

[13] Hao E., Schatz G.C. (2004). Electromagnetic fields around silver nanoparticles and dimers. J. Chem. Phys..

[14] Sivashanmugan K., Liao J., Liu B.H., Yao C., Luo S. (2015). Ag nanoclusters on ZnO nanodome array as hybrid SERS-active substrate for trace detection of malachite green. Sens. Actuators B Chem..

[15] Hrelescu C., Sau T.K., Rogach A.L., Jäckel F., Feldmann J. (2009). Single gold nanostars enhance Raman scattering. Appl. Phys. Lett..

[16] Yang Y., Shi J., Tanaka T., Nogami M. (2007). Self-assembled silver nanochains for surface-enhanced Raman scattering. Langmuir.

[17] Wang Y., Ruan W., Zhang J., Yang B., Xu W., Zhao B., Lombardi J.R. (2009). Direct observation of surface-enhanced Raman scattering in ZnO nanocrystals. J. Raman Spectrosc..

[18] Vlasko-Vlasov V., Joshi-Imre A., Bahns J.T., Chen L., Ocola L., Welp U. (2010). Liquid cell with plasmon lenses for surface enhanced Raman spectroscopy. Appl. Phys. Lett..

[19] Rycenga M., Kim M.H., Camargo P.H., Cobley C., Li Z., Xia Y. (2009). Surface-enhanced Raman scattering: Comparison of three different molecules on single-crystal nanocubes and nanospheres of silver. J. Phys. Chem. A.

[20] Lin J., Wang J., Xu C., Zeng Y., Chen Y., Li L., Huang Z., Li B., Chen R. (2017). Differentiation of digestive system cancers by using serum protein-based surface-enhanced Raman spectroscopy. J. Raman Spectrosc..

[21] Murdoch B.J., Portoles J.F., Tardio S., Barlow A.J., Fletcher I.W., Cumpson P.J. (2016). Visible wavelength surface-enhanced Raman spectroscopy from In-InP nanopillars for biomolecule detection. Appl. Phys. Lett..

[22] Jiang L., Li X., Wang A., Huang H., Feng J. (2017). L-Arginine-assisted one-pot synthesis of hierarchical Ag_1_Pt_2_ nanocorallines for surface-enhanced Raman spectroscopy. J. Colloid Interface Sci..

[23] Wu L., Xiao X., Chen K., Yin W., Li Q., Wang P., Lu Z., Ma J., Han H. (2017). Ultrasensitive SERS detection of Bacillus thuringiensis special gene based on Au@Ag NRs and magnetic beads. Biosens. Bioelectron..

[24] Boardman A.K., Wong W.S., Premasiri W.R., Ziegler L.D., Lee J.C., Miljkovic M., Klapperich C.M., Sharon A., Sauer-Budge A.F. (2016). Rapid detection of bacteria from blood with surface-enhanced Raman spectroscopy. Anal. Chem..

[25] Kim K., Kim K.L., Choi J., Shin D., Shin K.S. (2011). Effect of volatile organic chemicals on surface-enhanced Raman scattering of 4-aminobenzenethiol on Ag: Comparison with the potential dependence. Phys. Chem. Chem. Phys..

[26] Zeng Z., Tang D., Liu L., Wang Y., Zhou Q., Su S., Hu D., Han B., Jin M., Ao X. (2016). Highly reproducible surface-enhanced Raman scattering substrate for detection of phenolic pollutants. Nanotechnology.

[27] Chen Z., Qiu L., Tian Y., Lee Y., Hou X., Wu L. (2017). Surface-enhanced Raman scattering using monolayer graphene-encapsulated Ag nanoparticles as a substrate for sensitive detection of 2, 4, 6-trinitrotoluene. Anal. Methods.

[28] Mbah J., Moorer K., Pacheco Londoño L., Hernandez Rivera S., Cruz G. (2013). A rapid technique for synthesis of metallic nanoparticles for surface enhanced Raman spectroscopy. J. Raman Spectrosc..

[29] Nuntawong N., Eiamchai P., Wong-ek B., Horprathum M., Limwichean K., Patthanasettakul V., Chindaudom P. (2013). Shelf time effect on SERS effectiveness of silver nanorod prepared by OAD technique. Vacuum.

[30] Driskell J.D., Shanmukh S., Liu Y., Chaney S.B., Tang X., Zhao Y., Dluhy R.A. (2008). The use of aligned silver nanorod arrays prepared by oblique angle deposition as surface enhanced Raman scattering substrates. J. Phys. Chem. C.

[31] Chaney S.B., Shanmukh S., Dluhy R.A., Zhao Y. (2005). Aligned silver nanorod arrays produce high sensitivity surface-enhanced Raman spectroscopy substrates. Appl. Phys. Lett..

[32] Sun K., Meng G., Huang Q., Zhao X., Zhu C., Huang Z., Qian Y., Wang X., Hu X. (2013). Gap-tunable Ag-nanorod arrays on alumina nanotip arrays as effective SERS substrates. J. Mater. Chem. C.

[33] Gu G.H., Suh J.S. (2010). Silver nanorods used to promote SERS as a quantitative analytical tool. J. Raman Spectrosc..

[34] Huang Z., Meng G., Huang Q., Chen B., Zhu C., Zhang Z. (2013). Large-area Ag nanorod array substrates for SERS: AAO template-assisted fabrication, functionalization, and application in detection PCBs. J. Raman Spectrosc..

[35] Barranco A., Borras A., Gonzalez-Elipe A.R., Palmero A. (2016). Perspectives on oblique angle deposition of thin films: From fundamentals to devices. Prog. Mater. Sci..

[36] He Y., Fu J., Zhao Y. (2014). Oblique angle deposition and its applications in plasmonics. Front. Phys..

[37] Zhou Q., Li Z., Yang Y., Zhang Z. (2008). Arrays of aligned, single crystalline silver nanorods for trace amount detection. J. Phys. D Appl. Phys..

[38] Zhou Q., He Y., Abell J., Zhang Z., Zhao Y. (2011). Optical Properties and Surface Enhanced Raman Scattering of L-Shaped Silver Nanorod Arrays. J. Phys. Chem. C.

[39] Zhou Q., Zhang X., Huang Y., Li Z., Zhao Y., Zhang Z. (2012). Enhanced surface-enhanced Raman scattering performance by folding silver nanorods. Appl. Phys. Lett..

[40] Jen Y.J., Chan S., Huang J.W., Jheng C.Y., Liu W.C. (2015). Self-Shadowing Deposited Pure Metal Nanohelix Arrays and SERS Application. Nanoscale Res. Lett..

[41] Zhou Q., He Y., Abell J., Zhang Z., Zhao Y. (2011). Surface-enhanced Raman scattering from helical silver nanorod arrays. Chem. Commun..

[42] Liu Y., Chu H.Y., Zhao Y. (2010). Silver nanorod array substrates fabricated by oblique angle deposition: Morphological, optical, and SERS characterizations. J. Phys. Chem. C.

[43] Chu H., Huang Y., Zhao Y. (2008). Silver nanorod arrays as a surface-enhanced Raman scattering substrate for foodborne pathogenic bacteria detection. Appl. Spectrosc..

[44] Marotta N.E., Bottomley L.A. (2010). Surface-enhanced Raman scattering of bacterial cell culture growth media. Appl. Spectrosc..

[45] Shanmukh S., Jones L., Driskell J., Zhao Y., Dluhy R., Tripp R.A. (2006). Rapid and sensitive detection of respiratory virus molecular signatures using a silver nanorod array SERS substrate. Nano Lett..

[46] Hoang V., Tripp R.A., Rota P., Dluhy R.A. (2010). Identification of individual genotypes of measles virus using surface enhanced Raman spectroscopy. Analyst.

[47] Xiao C., Cao Z., Deng J., Huang Z., Xu Z., Fu J., Yobas L. (2014). Microfluidic-based metal enhanced fluorescence for capillary electrophoresis by Ag nanorod arrays. Nanotechnology.

[48] Leverette C.L., Villa-Aleman E., Jokela S., Zhang Z., Liu Y., Zhao Y., Smith S.A. (2009). Trace detection and differentiation of uranyl (VI) ion cast films utilizing aligned Ag nanorod SERS substrates. Vib. Spectrosc..

[49] Ma L., Huang Y., Hou M., Xie Z., Zhang Z. (2015). Silver nanorods wrapped with ultrathin Al_2_O_3_ layers exhibiting excellent SERS sensitivity and outstanding SERS stability. Sci. Rep..

[50] Bachenheimer L., Elliott P., Stagon S., Huang H. (2014). Enhanced thermal stability of Ag nanorods through capping. Appl. Phys. Lett..

[51] Bao L., Mahurin S.M., Dai S. (2004). Controlled Layer-By-Layer formation of ultrathin TiO_2_ on silver island films via a surface sol-gel method for surface-enhanced Raman scattering measurement. Anal. Chem..

[52] John J.F., Mahurin S., Dai S., Sepaniak M.J. (2010). Use of atomic layer deposition to improve the stability of silver substrates for in situ, high-temperature SERS measurements. J. Raman Spectrosc..

[53] Im H., Lindquist N.C., Lesuffleur A., Oh S. (2010). Atomic layer deposition of dielectric overlayers for enhancing the optical properties and chemical stability of plasmonic nanoholes. ACS Nano.

[54] Du P., Ma L., Cao Y., Li D., Liu Z., Wang Z., Sun Z. (2014). Stable Ag@oxides nanoplates for surface-enhanced Raman spectroscopy of amino acids. ACS Appl. Mater. Interfaces.

[55] Wang X., Wang Y., Yang J., Xing X., Li J., Wang L. (2009). Evidence of significant covalent bonding in Au (CN)^2−^. J. Am. Chem. Soc..

[56] Ma L., Huang Y., Hou M., Li J., Xie Z., Zhang Z. (2015). Pinhole-containing, subnanometer-thick Al_2_O_3_ shell-coated Ag nanorods as practical substrates for quantitative surface-enhanced Raman scattering. J. Phys. Chem. C.

[57] Ma L., Huang Y., Hou M., Xie Z., Zhang Z. (2015). Ag nanorods coated with ultrathin TiO_2_ shells as stable and recyclable SERS substrates. Sci. Rep..

[58] Song C., Chen J., Abell J.L., Cui Y., Zhao Y. (2011). Ag-SiO_2_ core-shell nanorod arrays: Morphological, optical, SERS, and wetting properties. Langmuir.

[59] Ma L., Huang Y., Hou M., Li J., Zhang Z. (2016). Pinhole effect on the melting behavior of Ag@Al_2_O_3_ SERS substrates. Nanoscale Res. Lett..

[60] Ma L., Wu H., Huang Y., Zou S., Li J., Zhang Z. (2016). High-performance real-time SERS detection with recyclable ag nanorods@HfO_2_ substrates. ACS Appl. Mater. Interfaces.

[61] Hou M., Huang Y., Ma L., Zhang Z. (2016). Quantitative analysis of single and mix food antiseptics basing on SERS spectra with PLSR method. Nanoscale Res. Lett..

[62] Yang K., Liu Y., Hsu T., Juang M. (2010). Strategy to improve stability of surface-enhanced Raman scattering-active Ag substrates. J. Mater. Chem..

[63] Mahurin S.M., Bao L., Dai S. (2006). Controlled layer-by-layer formation of ultrathin oxide films on silver island films for surface-enhanced Raman scattering measurement. Isr. J. Chem..

[64] Huang Y., Ma L., Hou M., Li J., Xie Z., Zhang Z. (2016). Hybridized plasmon modes and near-field enhancement of metallic nanoparticle-dimer on a mirror. Sci. Rep..

[65] George S.M. (2010). Atomic layer deposition: An overview. Chem. Rev..

[66] Puurunen R.L. (2005). Surface chemistry of atomic layer deposition: A case study for the trimethylaluminum/water process. J. Appl. Phys..

[67] Guziewicz E., Kowalik I.A., Godlewski M., Kopalko K., Osinniy V., Wójcik A., Yatsunenko S., Łusakowska E., Paszkowicz W., Guziewicz M. (2008). Extremely low temperature growth of ZnO by atomic layer deposition. J. Appl. Phys..

[68] Muraki N. (2014). In situ monitoring of thermal crystallization of ultrathin Tris(8-Hydroxyquinoline) aluminum films using surface-enhanced Raman scattering. Appl. Spectrosc..

[69] Formo E.V., Wu Z., Mahurin S.M., Dai S. (2011). In situ high temperature surface enhanced Raman spectroscopy for the study of interface phenomena: Probing a solid acid on alumina. J. Phys. Chem. C.

[70] Li X., Lee J., Blinn K.S., Chen D., Yoo S., Kang B., Bottomley L.A., El-Sayed M.A., Park S., Liu M. (2014). High-temperature surface enhanced Raman spectroscopy for *in situ* study of solid oxide fuel cell materials. Energ. Environ. Sci..

[71] Liu M., Xiang R., Cao W., Zeng H., Su Y., Gui X., Wu T., Maruyama S., Tang Z. (2014). Is it possible to enhance Raman scattering of single-walled carbon nanotubes by metal particles during chemical vapor deposition?. Carbon.

[72] An J., Tang B., Zheng X., Zhou J., Dong F., Xu S., Wang Y., Zhao B., Xu W. (2008). Sculpturing effect of chloride ions in shape transformation from triangular to discal silver nanoplates. J. Phys. Chem. C.

[73] Song C., Abell J.L., He Y., Murph S.H., Cui Y., Zhao Y. (2012). Gold-modified silver nanorod arrays: Growth dynamics and improved SERS properties. J. Mater. Chem..

[74] Hong B.H., Bae S.C., Lee C., Jeong S., Kim K.S. (2001). Ultrathin single-crystalline silver nanowire arrays formed in an ambient solution phase. Science.

[75] Bian Z., Zhu J., Cao F., Lu Y., Li H. (2009). In situ encapsulation of Au nanoparticles in mesoporous core-shell TiO_2_ microspheres with enhanced activity and durability. Chem. Commun..

[76] Dunnill C.W., Parkin I.P. (2011). Nitrogen-doped TiO_2_ thin films: Photocatalytic applications for healthcare environments. Dalton Trans..

[77] Li Y., Sasaki T., Shimizu Y., Koshizaki N. (2008). Hexagonal-close-packed, hierarchical amorphous TiO_2_ nanocolumn arrays: Transferability, enhanced photocatalytic activity, and superamphiphilicity without UV irradiation. J. Am. Chem. Soc..

[78] Zhao Y., Sun L., Xi M., Feng Q., Jiang C., Fong H. (2014). Electrospun TiO_2_ nanofelt surface-decorated with Ag nanoparticles as sensitive and UV-cleanable substrate for surface enhanced Raman scattering. ACS Appl. Mater. Interfaces.

[79] Hirakawa T., Kamat P.V. (2005). Charge separation and catalytic activity of Ag@TiO_2_ core-shell composite clusters under UV-irradiation. J. Am. Chem. Soc..

[80] Bao Z.Y., Liu X., Dai J., Wu Y., Tsang Y.H., Lei D.Y. (2014). In situ SERS monitoring of photocatalytic organic decomposition using recyclable TiO_2_-coated Ag nanowire arrays. Appl. Surf. Sci..

[81] Andrievskaya E.R., Lopato L.M. (2001). Phase equilibria in the Hafnia-Yttria-Lanthana system. J. Am. Ceram. Soc..

[82] Zhang X., Zhao J., Whitney A.V., Elam J.W., Van Duyne R.P. (2006). Ultrastable substrates for surface-enhanced Raman spectroscopy: Al_2_O_3_ overlayers fabricated by atomic layer deposition yield improved anthrax biomarker detection. J. Am. Chem. Soc..

[83] Allara D.L., Nuzzo R.G. (1985). Spontaneously organized molecular assemblies. 1. Formation, dynamics, and physical properties of n-alkanoic acids adsorbed from solution on an oxidized aluminum surface. Langmuir.

[84] Cheng H., Huan S., Wu H., Shen G., Yu R. (2009). Surface-enhanced Raman spectroscopic detection of a bacteria biomarker using gold nanoparticle immobilized substrates. Anal. Chem..

[85] Cowcher D.P., Xu Y., Goodacre R. (2013). Portable, quantitative detection of *Bacillus* bacterial spores using surface-enhanced Raman scattering. Anal. Chem..

[86] Sun F., Ella-Menye J., Galvan D.D., Bai T., Hung H., Chou Y., Zhang P., Jiang S., Yu Q. (2015). Stealth surface modification of surface-enhanced Raman scattering substrates for sensitive and accurate detection in protein solutions. ACS Nano.

[87] Li J., Li S., Anema J.R., Yang Z., Huang Y., Ding Y., Wu Y., Zhou X., Wu D., Ren B. (2011). Synthesis and characterization of gold nanoparticles coated with ultrathin and chemically inert dielectric shells for SHINERS applications. Appl. Spectrosc..

[88] Solovyeva E.V., Myund L.A., Dem Yanchuk E.M., Makarov A.A., Denisova A.S. (2013). Adsorption of acridine on silver electrode: SERS spectra potential dependence as a probe of adsorbate state. J. Mol. Struct..

[89] Gao J., Guo L., Wu J., Feng J., Wang S., Lai F., Xie J., Tian Z. (2014). Simple and sensitive detection of cyanide using pinhole shell-isolated nanoparticle-enhanced Raman spectroscopy. J. Raman Spectrosc..

[90] Würgler F.E., Schlatter J., Maier P. (1992). The genotoxicity status of sorbic acid, potassium sorbate and sodium sorbate. Mutat. Res. Lett..

[91] Tremblay G.C., Qureshi I.A. (1993). The biochemistry and toxicology of benzoic acid metabolism and its relationship to the elimination of waste nitrogen. Pharmacol. Ther..

[92] Wilson S.C., Brasel T.L., Martin J.M., Wu C., Andriychuk L., Douglas D.R., Cobos L., Straus D.C. (2005). Efficacy of chlorine dioxide as a gas and in solution in the inactivation of two trichothecene mycotoxins. Int. J. Toxicol..

[93] Thakur B.R., Singh R.K., Arya S.S. (1994). Chemistry of sorbates—A basic perspective. Food Rev. Int..

[94] Zhang W. (2010). Automotive fuels from biomass via gasification. Fuel Process. Technol..

[95] Menezes H.C., Amorim L.C., Cardeal Z.L. (2013). Sampling and analytical methods for determining VOC in air by biomonitoring human exposure. Crit. Rev. Environ. Sci. Technol..

[96] Yang L., Ma L., Chen G., Liu J., Tian Z.Q. (2010). Ultrasensitive SERS detection of TNT by imprinting molecular recognition using a new type of stable substrate. Chem.-Eur. J..

[97] Demeritte T., Kanchanapally R., Fan Z., Singh A.K., Senapati D., Dubey M., Zakar E., Ray P.C. (2012). Highly efficient SERS substrate for direct detection of explosive TNT using popcorn-shaped gold nanoparticle-functionalized SWCNT hybrid. Analyst.

[98] Park K.J., Wu C., Mercer-Smith A.R., Dodson R.A., Moersch T.L., Koonath P., Pipino A.C., Lu H., Yang Y., Sapirstein V.S. (2015). Raman system for sensitive and selective identification of volatile organic compounds. Sens. Actuators B Chem..

[99] Mosier-Boss P.A., Lieberman S.H. (2003). Detection of volatile organic compounds using surface enhanced Raman spectroscopy substrates mounted on a thermoelectric cooler. Anal. Chim. Acta.

[100] Pearman W.F., Fountain A.W. (2006). Classification of chemical and biological warfare agent simulants by surface-enhanced Raman spectroscopy and multivariate statistical techniques. Appl. Spectrosc..

[101] Stuart D.A., Biggs K.B., Van Duyne R.P. (2006). Surface-enhanced Raman spectroscopy of half-mustard agent. Analyst.

[102] Su Q., Ma X., Dong J., Jiang C., Qian W. (2011). A reproducible SERS substrate based on electrostatically assisted APTES-functionalized surface-assembly of gold nanostars. ACS Appl. Mater. Interfaces.

[103] Bizzarri A.R., Cannistraro S. (2007). SERS detection of thrombin by protein recognition using functionalized gold nanoparticles. Nanomed. Nanotechnol. Biol. Med..

[104] Green M., Liu F., Cohen L., Köllensperger P., Cass T. (2006). SERS platforms for high density DNA arrays. Faraday Discuss..

[105] Zhang X., Dai Z., Si S., Zhang X., Wu W., Deng H., Wang F., Xiao X., Jiang C. (2017). Ultrasensitive SERS substrate integrated with uniform subnanometer scale “hot spots” created by a graphene spacer for the detection of mercury ions. Small.

[106] Ouyang L., Hu Y., Zhu L., Cheng G.J., Irudayaraj J. (2017). A reusable laser wrapped graphene-Ag array based SERS sensor for trace detection of genomic DNA methylation. Biosens. Bioelectron..

[107] Wang X., Zhu C., Huang Z., Hu X., Zhu X. (2016). In situ synthesis of pristine-graphene/Ag nanocomposites as highly sensitive SERS substrates. RSC Adv..

[108] Liang X., Wang Y., You T., Zhang X., Yang N., Wang G., Yin P. (2017). Interfacial synthesis of three-dimensional hierarchical MoS_2_-NS@Ag-NPs nanocomposites as SERS nanosensor for ultrasensitive thiram detection. Nanoscale.

[109] Jiang S., Guo J., Zhang C., Li C., Wang M., Li Z., Gao S., Chen P., Si H., Xu S. (2017). A sensitive, uniform, reproducible and stable SERS substrate has been presented based on MoS_2_@Ag nanoparticles@pyramidal silicon. RSC Adv..

